# Walking barefoot vs. with minimalist footwear – influence on gait in younger and older adults

**DOI:** 10.1186/s12877-020-1486-3

**Published:** 2020-03-04

**Authors:** Evi Petersen, Astrid Zech, Daniel Hamacher

**Affiliations:** 1 Institute of Sports, Physical Education and Outdoor Life, University of South-Eastern Norway, Bø and Telemark, Norway; 2 Institute of Sports Science, Friedrich-Schiller University of Jena, Jena, Thuringia Germany

**Keywords:** Elderly, Community-dwelling, Inertial sensors, Leguano, Minimalist shoes, Barefoot

## Abstract

**Background:**

In recent years, minimalist footwear has been increasingly promoted for its use in sportive and recreational activities. These shoes are considered to function naturally like barefoot walking while providing a protective surface. Despite a growing popularity of these shoes in the older population, little is known about the influence of minimalist footwear on gait patterns. This study investigated whether overground walking with minimalist shoes is comparable to barefoot walking regarding gait stability and variability parameters.

**Methods:**

In a randomized within-subject study design, 31 healthy younger (29 ± 4 years) and 33 healthy community-dwelling older adults (71 ± 4 years) volunteered. Participants walked on flat ground, once barefoot and once with minimalist shoes. Gait variability of minimum toe clearance (MTC), stride length, stride time, and local dynamic gait stability were analysed.

**Results:**

The results for both age groups showed significant condition effects (minimalist shoes vs. barefoot walking) for the outcomes of local dynamic stability (*p* = .013), MTC variability (*p* = .018), and stride length variability (*p* < .001) indicating increased local dynamic stability and decreased gait variability during the minimalist shoe condition. Group effects (young vs. older adults) were detected in all gait outcomes.

**Conclusion:**

Walking with minimalist shoes appeared to be associated with better gait performance than walking barefoot in both age groups. Thus, walking with minimalist shoes is not similar to barefoot walking. With respect to reducing the risk of falling, we suggest that minimalist shoes could be an alternative to barefoot walking or a transition option between shoes to barefoot for older adults.

## Background

Bipedal gait is one of the most fundamental sensorimotor tasks performed every day [1]. Especially in older adults, a well-functioning gait pattern is recognized to be essential for autonomous participation in daily life [2]. With an increase in age, however, deficiencies in gait frequently evolve while the risk of falling increases [3]. Due to degenerative processes of the neuromuscular system and other age-related adaptations, older adults typically exhibit the following gait characteristics: (i) a wider stance and extended bipedal ground contact with shorter steps [4] as well as (ii) a diminished swing phase [5]. Besides internal factors, evidence reveals that footwear as an external criterion has a significant impact on the gait pattern [6, 7].

Footwear has been implicated as a factor in falls, which again is a crucial issue affecting health and quality of life in older adults [8, 9]. In relation to this, older adults are often advised to wear shoes with low heels and firm slip-resistant soles [10]. McKeon et al. [11] noted that permanent support to the foot might result in degenerative efficiency in foot muscles and sensitivity, and therefore carry a potential of adverse effects on the gait pattern. Accordingly, [11] suggest that walking barefoot is less restricting for motion control, which increases the sensitivity of the sensory mechanisms and activates the foot and lower leg muscles. Both sensory feedback sensitivity [12] and increased foot strength [13] showed to improve balance in older adults, and are therefore significant predictors in the prevention of falls. Thus, barefoot walking might result in beneficial effects on sensorimotor control.

In recent years, minimalist footwear (characterized by light weight, high flexibility and absence of cushioning material) has been increasingly promoted for its use in sportive and recreational activities. Wear of such footwear has been shown to be closely related to barefoot running conditions [14–16]. Therefore, it considered to function naturally like barefoot walking while providing a protective surface. Despite the growing popularity of these shoes in the older population, little is known about the influence of minimalist footwear on gait patterns and performance [17, 18]. In relation to this, local dynamic gait stability, as well as gait variability measures, were associated with gait performance and the likelihood of falling [19, 20]. More specifically, measures of local dynamic gait stability (LDS) are capable of distinguishing between cohorts of younger and older adults, while lower levels of LDS are associated with a higher risk of falling [21]. Furthermore, the minimum foot clearance (MFC) variability is a promising gait variable that can predict the risk of falling, with greater variabilities of MFC indicating a higher risk of falling [22].

In conclusion, walking with minimalist shoes seems to merge the positive effects of barefoot walking while providing a protective surface. However, the effects on the gait pattern were not sufficiently studied yet. Consequently, the primary aim of our study was to investigate if overground walking with minimalist shoes is comparable to barefoot walking regarding gait stability and variability. A study by Smith et al. [23] showed that minimalist shoes provided better overall and anterior-posterior static balance than walking barefoot. Additionally, [17] suggested that wearing minimalist shoes could provide benefits of barefoot walking in respect to fall prevention, while simultaneously offering some protection and support to the feet. Therefore, we predicted that overground walking with minimalist shoes is associated with higher gait stability and lower gait variability. We furthermore analyzed whether this relationship is similar in young and older adults.

## Methods

### Participants

In a randomized within-subject study design, gait data of 31 healthy younger (17 female, 14 male, age: 29 ± 4 years; BMI: 23 ± 2) and 33 healthy community-dwelling older (20 female, 13 male, age: 71 ± 4 years; BMI: 27 ± 4) participants were collected. We advertised in the local newspaper and at a local sport club to recruit the participants of this study.

Inclusion criteria were the age of ≤35 years for the younger group and ≥ 65 years for the older group. Participants had to be able to walk throughout 5 minutes without the need to pause or the use of assisting equipment. Additionally, participants had to be unfamiliar with regular barefoot walking or walking with minimalist shoes. Any self-reported motor-functional impairments that could affect gait performance, such as acute musculoskeletal disorders or neurological diseases, led to exclusion from the study. All subjects provided their written informed consent to their voluntary participation in this study as well as their allowance for publication, which has been approved by the local ethics committee (protocol no. FSV 16/13) and followed the principles of the Helsinki Declaration.

### Testing procedure

To capture kinematic data, wireless inertial sensors (MTw2, Xsens Technologies B.V., Enschede, The Netherlands, range of measurement of angular velocity: ±1200 deg/s, sampling rate: 100 Hz) were attached to the participants’ right forefeet using tape. The participants were block randomized to the different test condition sequences by using the computer-based program *Research Randomizer* (https://www.randomizer.org/). Further, participants were asked to walk at their preferred walking pace back and forth on a 25 m track inside a sports hall with flat ground. The following test conditions were performed in a randomized and balanced order: 1) barefoot walking and 2) walking with minimalist shoes (leguano classic, leguano GmbH, Buchholz, Germany; material: 53% polyamide, 38% lyocell, 7% polypropylene, 2% elastane, Sole: 100% LIFOLIT®). Participants walked for 3 minutes under each condition. The resting time between the test conditions was 5 minutes. Prior to each of the testing conditions, participants walked for approximately 1 minute back and forth the track to familiarize themselves with the corresponding test condition and to improve reliability [24].

### Data analysis and statistics

The data analysis included gait variability measures as well as the local dynamic gait stability for which the largest Lyapunov exponent (*λ*) was calculated. We removed the first strides (from the start to the first turn after 25 m) of each trial as well as the last strides (from the last turn to the stop) to avoid possible transients, e.g. to analyze steady-state gait [25]. Furthermore, we removed the first 2.5 m of each section between two successive turns to exclude the acceleration periods after turning [26]. To account for possible deceleration periods before turning, the last 2.5 m were excluded from the subsequent analysis as well. At least 50 strides should be analyzed for gait variability measures [27]. In this study, the first 80 valid strides within each trial were analyzed.

To calculate the gait measures a) stride length, b) stride time and c) minimum toe clearance (MTC), we used a published algorithm [25]. As gait variability measures, the intraindividual standard deviations of each gait measure were analysed. To quantify *λ,* we time-normalized the three-dimensional angular velocity data of the 80 valid strides to 8000 samples. To reconstruct the state space, we applied the embedding approach. The time delay (9 samples) and the embedded dimension (dE = 6) were determined, using the first minimum mutual information [28] and the global false nearest neighbors method, respectively [29]. The *λ* was determined using the Rosensteins algorithm [30], which we implemented in MATLAB (version 2016a, TheMathWorks BV, Natrick, USA). The Euclidean distance of each nearest neighbor state space was tracked while the mean of the logarithm of the divergence curve was calculated. *λ* is defined as the slope (linear fit) through 0–0.5 strides. The implementation was validated previously [25]. Using IBM SPSS Statistics (V 21.0) for all statistical procedures, a two factor (condition: barefoot and minimalist footwear; age: younger and older adults) variance analysis with repeated measures was applied to identify differences across the two conditions.

## Results

**Fig. 1:**
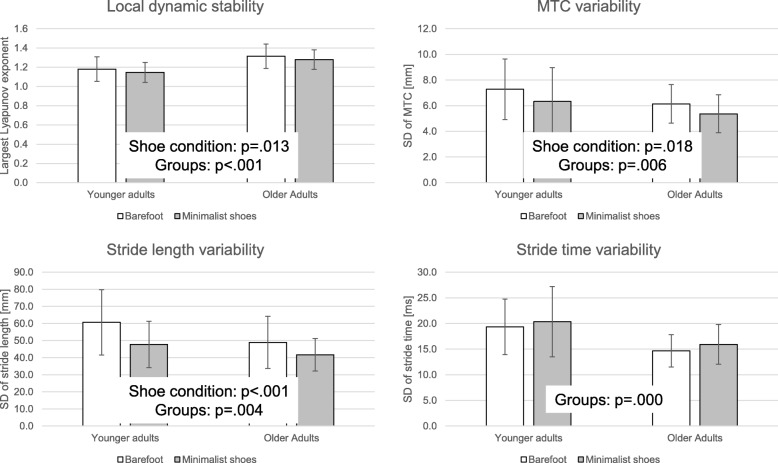
Gait stability and variability in younger and older adults – barefoot vs. minimalist shoes

As Fig. 1 illustrates, the results show significant condition effects (minimalist shoes vs. barefoot walking) for the outcomes of local dynamic stability (*p* = .013, partial η^2^ = 0.10), MTC variability (*p* = .018, partial η^2^ = 0.09), and stride length variability (*p* < .001, partial η^2^ = 0.26). These outcomes indicate increased local dynamic stability (lower largest Lyapunov exponent) and decreased gait variability during the minimalist shoe condition. There was no condition effect on stride time variability. Group effects (young vs. older adults) in all gait outcomes (LDS: *p* < .001, partial η^2^ = 0.30; MTC variability: *p* = .006, partial η^2^ = 0.12; stride length variability: *p* = .004, partial η^2^ = 0.13; stride time variability: *p* = <.001, partial η^2^ = 0.26) were found. These effects indicate better LDS in younger participants and, surprisingly, better (lower) gait variability in older participants. There was no significant interaction effect between the walking condition and the group condition.

## Discussion

To the best of our knowledge, this is the first study that investigated whether overground walking with minimalist shoes is comparable to barefoot walking regarding gait stability and variability parameters in both younger and older adults. Current research discussed the use of minimalist shoes as an alternative to barefoot running or a method of transitioning between shod and barefoot running for athletes [23]. Overall and valid for both age groups, our results suggest that walking with minimalist shoes is associated with better gait stability and variability measures, thus, with a lower risk of falling [21, 22]. Consequently, walking with minimalist shoes is not similar to barefoot walking, and the positive effects of barefoot walking [11] are not necessarily generalizable to walking with minimalist shoes.

In order to reduce the risk of falling, we suggest that walking training with minimalist shoes could be an alternative to barefoot walking or a transition option between shoes to barefoot for older adults. Our proposition is in line with [17], who state that wearing minimalist shoes could provide benefits of barefoot walking in respect of fall prevention, while simultaneously offering some protection and support to the feet. Further, wearing minimalist shoes might help older adults to overcome different barriers, which are associated with walking barefoot. Often reported barriers are being ashamed of one’s own feet, fear of falling or fear of instability as well as the sense of having cold feet [10, 31].

Research on younger adults and children [15] showed that barefoot training can result in long-term outcomes, such as reduced ankle dorsiflexion at foot strike. That points to the prospect that wearing minimalist footwear regularly could have a positive impact on reducing the risk of falling. In one of their recent studies, Franklin et al. [32] investigated whether wearing minimalist shoes daily for 4 months could lead to improvements in balance and foot strength, which are both critical variables concerning a functional gait pattern. Preliminary results show that training with minimalist shoes can improve both parameters and thereby emphasize the potential of minimalist shoe use to reduce the risk of falling. That is in line with our results, which show that the use of minimalist shoes improved gait stability compared to barefoot walking.

We found less LDS in older adults compared to younger adults. This outcome has been frequently reported [19–21]. In contrast, it is surprising that older adults showed better (lower) gait variability than younger adults. A recent study by Hamacher et al. [33] reported similar outcomes. In their study, younger and older participants walked in their own shoes; however, the same system and algorithms were applied [33]. In the current study, younger adults show a much higher MTC, stride length and stride time variability during barefoot walking and walking with minimalist shoes compared to the younger participants of [33], walking in their own shoes. The older adults of the current study, on the other hand, show only a minor increase in gait variability compared to the older participants of the other study. We conclude that the group effect of the gait variability measures could be the result of younger adults adapting more quickly to an unfamiliar walking condition (walking barefoot / walking with minimalist shoes) to maintain local dynamic stability. This effect seems to be less pronounced in older adults. Since this is speculative, more research is needed to investigate the issue.

### Study limitations and future research implications

We want to acknowledge four main study limitations. First, this research involved healthy community-dwelling older adults. Therefore, our current findings may not apply to older adults with health issues or already existing impairments. Most older adults have a higher risk of falling when confronted with existing dysfunctions concerning the gait pattern [34]. This fact points to the potential of future research within clinical settings.

Second, the participants of this study did not walk in their own shoes. Implementing this third condition to the testing procedure could have revealed more knowledge of how minimalist shoes influence gait in comparison to normal shoes, which are worn in daily life. Additionally, it would have made our results even more comparable to other studies. However, this question was not subject of our current study. We also ran our tests for only one type of minimalist shoe. Considering the growth of diversity in products, we encourage future research to compare the influence of different minimalist footwear as well as to include aspects of practicability regarding minimalist shoes for older adults.

Third, the application side in this study was a gym, proving a flat ground. Thus, our findings are limited to overground walking on an even surface. According to Zurales et al. [35], however, uneven surfaces are strong predictors of falls among older adults. Additionally and in line with Li et al. [36], falls occurred more often outdoors than indoors among older adults. Thus, future research should consider testing on an uneven surface, for instance, outdoors.

Last, this study focused on short-term effects. We suggest that future research should target long-term effects of fall prevention training with minimalist shoes or extended periods of wearing minimalist shoes during daily activity. Certainly, a systematic overview of the existing literature would enrich this scope of research.

## Conclusion

This study demonstrates for walking in a straight line on flat ground that minimalist shoes, as compared to walking barefoot, have significantly different effects on gait and fall predicting parameters such as gait stability (LDS) and variability (MTC). Effects were observed for the group of younger as well as older adults. Walking with minimalist shoes was overall associated with better gait performance than walking barefoot. This finding hence demonstrates the potential of minimalist shoes as a means to prevent falls. We conclude that there is a need for future research to investigate the benefits of minimalist shoes for more complex walking tasks such as walking on uneven ground. Further, the long-term effects of minimalist usage need to be explored, particulary in relation to standard footwear. Simultaneously, we make a call for a systematic review of the literature concerned with minimalist footwear and the risk of falling.

## Data Availability

The dataset for this manuscript is not publicly available, however, requests to access the datasets can be directed to Evi Petersen (evi.petersen@usn.no).

## Abbreviations

LDS: Local dynamic gait stability
MFC: Minimum foot clearance
MTC: Minimum toe clearance
*λ*: Lyapunov exponent
